# Youth Perspectives on Generative AI and Its Use in Health Care

**DOI:** 10.2196/72197

**Published:** 2025-05-21

**Authors:** Christian Schaaff, Manvir Bains, Sophie Davis, Trinity Amalraj, Abby Frank, Marika Waselewski, Tammy Chang, Andrew Wong

**Affiliations:** 1 University of Michigan Medical School, Ann Arbor, MI, United States; 2 University of Notre Dame, Notre Dame, United States; 3 Michigan State University, East Lansing, United States; 4 Department of Family Medicine, University of Michigan Medical School, Ann Arbor, MI, United States; 5 Institute for Healthcare Policy and Innovation, University of Michigan, Ann Arbor, MI, United States; 6 Department of Internal Medicine, University of Michigan Medical School, 2800 Plymouth Road, Building #14, Room G100-22, Ann Arbor, MI, 48109-2800, United States, 1 7349364000

**Keywords:** generative artificial intelligence, medical informatics, adolescent health, health technology, young adult

## Abstract

A nationwide survey of youth aged 14 to 24 years on generative artificial intelligence (GAI) found that many youths are wary about the use of GAI in health care, suggesting that health professionals should acknowledge concerns about AI health tools and address them with adolescent patients as they become more pervasive.

## Introduction

Generative artificial intelligence (GAI) has become increasingly prevalent throughout health care, with GAI tools being applied to clinical decision support, medical documentation, patient-provider communication, and more [1,2]. GAI is unique in its widespread adoption by both the general public and technical professionals [3]. In particular, younger individuals are more likely to adopt GAI technologies [4]. In this nationwide qualitative survey, we characterized the attitudes of youth aged 14 to 24 years toward GAI technology. We then extracted potential implications of applying GAI to health care for adolescents and young adults, presenting our findings herein.

## Methods

### Ethical Considerations

This study was approved by the Institutional Review Board of the University of Michigan Medical School. Informed consent was obtained via the internet for all participants. All data were deidentified and protected by a National Institutes of Health Certificate of Confidentiality. Participants received US $1 per week for answering each week’s survey and a US $3 bonus if all questions were answered during the 8- to 12-week phase. US $5 was provided upon enrollment completion, which included a web-based demographic survey.

### Study Design

Study participants were respondents of MyVoice—a nationwide text message survey that collects perspectives from youth aged 14 to 24 years. Five open-ended questions related to general GAI use were texted to participants in March 2024 (Multimedia Appendix 1) [5,6]. Using content analysis, 2 investigators reviewed responses by question, developed a codebook, and independently applied codes. Discrepancies were resolved via discussion. Code frequency and demographic data, including age, sex, ethnicity, zip code, and socioeconomic status (defined as “low” if respondents ever used the Supplemental Nutrition Assistance Program [SNAP]), were summarized by using descriptive statistics [5]. Zip codes were aggregated into the geographic regions outlined by the American Community Survey [5]. Themes identified in the survey responses, including those specific to health care, were coded across all questions. Participants’ responses could count toward multiple categories.

## Results

Of 758 eligible youths, 624 (82.3%) responded to at least one question. On average, respondents were aged 20.3 (SD 2.6) years (Table 1). A majority (328/624, 52.6%) were female. About half (361/624, 57.9%) of respondents were White, 13.8% (86/624) were Black, and 13.6% (85/624) were Hispanic. Further, 25.7% (145/564) of respondents experienced low socioeconomic status (ie, current or previous use of the SNAP). Of 619 respondents (5 survey responses were excluded for being blank or nonsensical), 95.6% (592/619) endorsed prior knowledge of GAI, with 10% (62/619) sharing a positive opinion of GAI, 7.4% (46/619) expressing disapproval, and 82.6% (511/619) expressing neither; 31.3% (194/619) found GAI useful.

Survey response themes are summarized in Table 2. A majority of participants (474/619, 76.6%) endorsed use of GAI as a study aid, writing tool, or efficiency booster (Table 2). Among the 23.4% (145/619) of respondents who had not used GAI, frequently cited reasons included a lack of need or interest (46/145, 31.7%), ethical concerns (30/145, 20.7%), and authenticity concerns surrounding GAI (18/145, 12.4%). Notably, only 2.1% (10/474) expressed explicit dislike of GAI after use.

Across all survey responses, 10.6% (66/624) of respondents mentioned health applications, with 40.9% (27/66) citing specific health-related applications (eg, creating meal plans, designing exercise regimens, refining medical diagnoses, and improving health care systems) and 63.6% (42/66) expressing concerns. These concerns primarily focused on the need for human input in medical decision-making (12/42, 28.6%) and the importance of avoiding medical errors (8/42, 19%).

**Table 1.** Demographic characteristics of 624 youth respondents in the MyVoice survey on generative artificial intelligence.

Demographic variable	Respondents
Age (years), mean (SD)	20.3 (2.6)
Gender identity (respondents: n=624), n (%)	
Male	210 (33.7)
Female	328 (52.6)
Transgender	35 (5.6)
Nonbinary/other	51 (8.2)
Race (respondents: n=624), n (%)	
White	361 (57.9)
Black	86 (13.8)
Asian	103 (16.5)
Mixed race	48 (7.7)
Other race	26 (4.2)
Hispanic (respondents: n=624), n (%)	
Yes	85 (13.6)
No	539 (86.4)
Highest education level (respondents: n=623), n (%)	
Less than high school	123 (19.7)
High school graduate	79 (12.7)
Some college or technical school	236 (37.9)
Associate’s or technical degree	41 (6.6)
Bachelor’s degree or higher	144 (23.1)
Region (respondents: n=623), n (%)	
Midwest	203 (32.6)
Northeast	127 (20.4)
South	164 (26.3)
West	129 (20.7)
SNAP^a^ use (respondents: n=564), n (%)	
Current	51 (9.0)
Previous	94 (16.7)
Never	419 (74.3)

a SNAP: Supplemental Nutrition Assistance Program.

**Table 2.** Survey themes, responses, and representative quotations from the MyVoice survey on generative artificial intelligence (GAI).

Theme	Responses^a^, n (%)	Representative quotes
Current patterns of GAI usage		
Boosting efficiency	159 (27)	“Helping to automate tedious tasks”
Academics (school)	95 (16.1)	“Yes, primarily as a research tool for school”
Health-related activities	27 (4.6)	“I’ve heard of [GAI] being used…to [alert] Healthcare workers to potential outcomes.”
Meal ideas	9 (33.3)	“Make(s) it easier to find recipes for cooking”
Managing disabilities	4 (12.1)	“I am disabled, it helps me with tasks such as drafting emails (and where)…it matters how you come across.”
Exercise	3 (11.1)	“Basically a free personal trainer”
Concerns regarding GAI usage		
Misinformation	159 (28.8)	“[GAI] can blur the lines between reality and fiction.”
Job security	148 (26.8)	“I am concerned it is going to take jobs.”
Excessive dependence	92 (16.6)	“[GAI] can be a crutch, and make people overdependent on technology. It may also be used to stifle creativity…”
Medical concerns	3 (0.5)	“[GAI] can become dangerous when used in medical situations…”
Circumstances in which GAI should not be used		
School	180 (31.7)	“Writing academic papers or doing school assignments...”
Unethical activities	103 (18.2)	“It should NEVER be used to create images/videos/…of people without consent”
Supplanting human creativity	115 (20.3)	“AI shouldn’t be used for artistic purposes because art is intrinsically rooted in human emotion and experience”
Health care (generally)	40 (7.1)	“The thought of [GAI] in the medical field is unsettling”
Complex scenarios in health care	5 (0.9)	“Medicine/healthcare - too many components that I wouldn’t trust the machine to handle”
Health care because of the need for human input	12 (2.1)	“Healthcare industry jobs that require human consideration, life or death care I guess”
Concern for mistakes or biases in health care settings	8 (1.4)	“It should not be used to answer serious questions such as legal or medical advice since it can cause fatal errors.”
High-stakes situations in health care	5 (0.9)	“It’s simple for [GAI] to make a simple mistake that can result in someone’s life being on the line.”

a Survey responses were coded across all themes. A single response could count toward multiple categories.

## Discussion

Our study found that GAI adoption among American youth (77.2%) is high when compared to the general population (39.5%), which is consistent with previous research [7,8]. GAI use among adolescents and young adults ranged across many fields, including academics, health care, and daily efficiency. Despite the widespread GAI adoption among American youth, many remain uncomfortable with GAI use in health care settings; this sentiment’s prevalence among youth is similar to that among adult patients [9]. Respondents who discussed GAI in the context of health care primarily focused on concerns about medical decision-making and medical errors, highlighting the importance of transparency and disclosure when using GAI tools to treat adolescents and young adults.

Interestingly, the use of GAI platforms to address personal health concerns was not mentioned by participants in this study. This finding suggests that self-diagnosis is not currently a primary focus of youth who use GAI tools. Rather, youth more frequently voice concern with safeguarding medical processes against error and ensuring that human input is retained in medical decision-making.

In summary, youth remain wary of GAI applications in health care despite widespread GAI adoption for other use cases. Health care professionals can build trust and rapport with adolescents and young adults by acknowledging and addressing their concerns as GAI use becomes more prevalent in health care. Further research into meaningfully applying GAI to youth health is warranted to guide successful implementation of this novel technology.

## Supplementary material

10.2196/72197 Multimedia Appendix 1 Probing survey questions with corresponding response rates from the MyVoice survey on generative artificial intelligence.

## Abbreviations

GAI: generative artificial intelligence
SNAP: Supplemental Nutrition Assistance Program
