# Supplementary material for: Youth Perspectives on Generative AI and Its Use in Health Care
Source: J Med Internet Res. 2025 May 21;27:e72197. doi: 10.2196/72197 (PMC12118938; doi:10.2196/72197)
Supplement: Multimedia Appendix 1 [file jmir-v27-e72197-s001.docx]

**Supplemental Table: Probing Survey Questions with Corresponding Response Rate from the MyVoice Survey on Generative Artificial Intelligence**

| Survey Question | Responses (%) |
| --- | --- |
| Generative Artificial Intelligence (like ChatGPT) uses algorithms to create new content, including audio, code, images, text, simulations, and videos. Have you heard of this? Tell us about it. | 619 (81.7) |
| Have you used Generative Artificial Intelligence (AI) for anything? Why or why not? | 605 (79.8) |
| What are ways that Generative AI could make (or has made) your life better? | 589 (77.7) |
| What concerns do you have about Generative AI? Why? | 553 (73.0) |
| In what situations do you think Generative AI should NOT be used? Why? | 567 (74.8) |
|  |  |
